# Query into Tuberculosis Infection Screening and Management among Pregnant Migrants, Europe

**DOI:** 10.3201/eid3203.251775

**Published:** 2026-03

**Authors:** Filippo Cioli Puviani, Amina Zaffagnini, Cristina Mazzi, Daniela Maria Cirillo, Francesco Di Gennaro, Ferenc Balázs Farkas, Lorenzo Guglielmetti, Yousra Kherabi, Heinke Kunst, Chiara Sepulcri, Christian Wejse, Laura Todaro, Tamara Ursini

**Affiliations:** University of Verona, Verona, Italy (F. Cioli Puviani); IRCCS Sacro Cuore Don Calabria Hospital, Negrar di Valpolicella, Italy (A. Zaffagnini, C. Mazzi, L. Guglielmetti, T. Ursini); Emerging Bacterial Pathogens Unit, IRCCS San Raffaele Scientific Institute, Milan, Italy (D.M. Cirillo, C. Sepulcri); University of Bari, Bari, Italy (F. Di Gennaro); Institute of Medical Microbiology, Faculty of Medicine, Semmelweis University, Budapest, Hungary (F.B. Farkas); Pediatric Center, Semmelweis University, Budapest (F.B. Farkas); Bichat-Claude Bernard Hospital, Assistance Publique-Hôpitaux de Paris, Université Paris Cité, Paris, France (Y. Kherabi); Université Paris Cité, Inserm, Paris (Y. Kherabi); Queen Mary & Barts Health Tuberculosis Centre, Blizard Institute, Faculty of Medicine & Dentistry, London, UK (H. Kunst); University of Genoa, Genoa, Italy (C. Sepulcri); Aarhus University Hospital, Aarhus, Denmark (C. Wejse); Center for Global Health, Aarhus University, Aarhus (C. Wejse); Provincial Hospital of Bolzano, Bolzano-Bozen, Italy (L. Todaro)

**Keywords:** Tuberculosis and other mycobacteria, bacteria, respiratory infections, TB, Mycobacteria tuberculosis, infection, pregnancy, migrants, prenatal care, antenatal care, screening, preventive treatment, Europe

## Abstract

Pregnant migrant women face increased tuberculosis vulnerability. We queried clinicians in Europe on *Mycobacterium tuberculosis* infection screening and management among pregnant migrants. Fewer than half reported routinely performing screening, and diagnostic and preventive practices varied widely. Those responses highlight substantial heterogeneity and uncertainty in current *M. tuberculosis* infection screening practices.

Tuberculosis (TB), caused by *Mycobacterium tuberculosis*, remains a major global health threat. The World Health Organization (WHO) European Region reported 172,300 TB cases and 22,500 deaths in 2023 (*1*,*2*). Migrants from high-incidence countries are disproportionately affected by TB (*3*). Pregnancy further increases vulnerability because of immunologic changes and healthcare barriers (*4*). Early identification of *M.* *tuberculosis* infection during pregnancy could represent an opportunity for prevention; however, evidence on the balance between potential benefits and risks remains limited (*5*). Current WHO recommendations restrict TB preventive treatment (TPT) during pregnancy to persons living with HIV and are largely derived from high-burden settings, which have limited applicability to migrant populations in Europe (*6*). Although drugs included in TPT regimens are used for treating TB during pregnancy, safety data remain limited (*6*). Given those gaps, we queried clinicians in Europe on *M.* *tuberculosis* infection screening and preventive practices for pregnant migrant women. 

We disseminated an online query during March 4–May 31, 2025 (Appendix 1; Appendix 2), to gather information on *M.* *tuberculosis* infection screening and management practices for pregnant migrants in Europe. The query was endorsed by the European Society of Clinical Microbiology and Infectious Diseases Study Group for Infections in Travelers and Migrants and Study Group for Mycobacterial Infections. We descriptively summarized responses.

A total of 101 professionals responded, 74.3% (75/101) of whom were infectious diseases specialists, and most worked in hospitals. Participants represented 20 different countries, most within the WHO European Region (Appendix 1 Table). Only 27.7% (28/101) reported routinely offering *M.* *tuberculosis* infection screening to pregnant migrants, but 36.6% (37/101) reported screening migrants who had specific risk factors, such as HIV infection, recent (<5 years) arrival from high-incidence countries, immunosuppression, or close contact with a TB case (Table). 

**Table:** Risk factors and barriers reported by respondents to a network query into TB infection screening and management among pregnant migrants, Europe, 2025*

Risk factor or barrier	No. (%) respondents
Risk factors considered when screening offered during pregnancy, n = 37†	
Recent TB contact	34 (91.9)
HIV infection	34 (91.9)
Immunosuppression	31 (83.8)
Recent migration, <5 y	22 (59.4)
Homelessness	12 (32.4)
Diabetes	7 (18.9)
Malnutrition	6 (16.2)
Risk factors considered when TB preventive treatment offered during pregnancy, n = 31†	
HIV infection	30 (96.8)
Recent TB contact	27 (87.1)
Immunosuppression	22 (71)
Recent migration, <5 y	11 (35.5)
Diabetes	7 (22.6)
Malnutrition	7 (22.6)
Homelessness	5 (16.1)
Perceived barriers to TB infection screening, n = 37†	
Chest radiograph issues‡	28 (75.7)
Patient adherence	11 (29.7)
Financial or healthcare system barriers	10 (27)
Guideline gaps	8 (21.6)
Perceived barriers to TB preventive treatment, n = 42†	
Patient-related factors	33 (78.6)
Healthcare provider–related factors	20 (47.6)
Guideline or policy gaps	16 (38.1)
Healthcare system barriers	12 (28.6)

*TB, tuberculosis. †Multiple responses were permitted. ‡Includes concerns regarding radiation exposure, which trimester radiographs can safely be administered, and patient acceptance of chest radiography during pregnancy.

Among screening tools, of participants who reported screening pregnant migrants for *M.* *tuberculosis* infection, 82.6% (57/69) reported using interferon-γ release assays (IGRAs), and 27.5% (19/69) used chest radiographs. Tuberculin skin testing was less commonly adopted, serving as the main diagnostic tool for only 24.6% (17/69). To diagnose *M. tuberculosis* infection, 60.9% (42/69) of respondents reported using a sequential diagnostic approach in which positive tuberculin skin test or IGRA result was followed by chest radiograph to rule out TB disease among IGRA-positive women. An additional 37.7% (26/69) used a similar approach but relied on clinical assessment instead of imaging to exclude TB disease. 

Approaches to TPT during pregnancy varied widely among respondents; 30.4% (21/69) reported routinely offering TPT to all pregnant migrants with diagnosed *M. tuberculosis* infection, 40.6% (28/69) did so only under specific conditions, and 23.2% (16/69) postponed treatment until after delivery. When TPT was initiated during pregnancy, most respondents started therapy as soon as *M. tuberculosis* infection diagnosis was made, regardless of fetal gestational age. The most common regimens were isoniazid plus rifampin or isoniazid monotherapy.

Participants reported barriers to *M. tuberculosis* infection screening and treatment in pregnant migrants; 53.6% (37/69) identified challenges in performing screening and 62.7% (42/67) reported difficulties in prescribing or ensuring adherence to TPT (Table). The most common reported barriers to screening included concerns about radiation exposure from chest radiographs, unclear protocols, and uncertainty about timing for safely administering chest radiographs during pregnancy. Additional issues for both screening and treatment included patient adherence, limited resources, and lack of clear guidelines (Table).

Regarding TPT, respondents reported the main difficulties were patient-related, such as language and cultural barriers and fears about gestational risks and side effects. Among healthcare provider–related concerns, reported difficulties included uncertainty about which stage of pregnancy is considered safe for starting TPT and lack of training. Participants also noted healthcare system challenges, such as fragmented follow-up pathways and limited availability of dedicated services. Estimated TPT adherence rates varied, and only one third of respondents estimated high (>80%) adherence.

Slightly more than one third (35.7%, 35/98) of respondents reported following specific guidelines for *M. tuberculosis* infection screening in pregnant women, predominantly international guidelines (*6*–*8*) and, to a lesser extent, national guidelines (*9*,*10*). However, only 23.2% (23/99) considered the available guidelines adequate. Guideline limitations included a lack of evidence specific to pregnancy, insufficient guidance on when and how to screen and treat pregnant women, and inconsistent national recommendations. Respondents noted training gaps, and one third of participants had received no specific training on *M. tuberculosis* infection screening and treatment. Respondents emphasized the need for additional resources, particularly standardized protocols, training, better access to guidelines, and the involvement of cultural mediators.

In this query among clinicians in Europe, respondents reported substantial heterogeneity in *M. tuberculosis* infection screening and TPT practices for pregnant migrants. Screening was most often restricted to women with specific risk factors, and diagnostic and preventive approaches varied widely across settings. Given the convenience sampling approach and the open-link dissemination strategy (response rates not assessable), the patterns described here should not be interpreted as representative of all settings in Europe. However, the responses to our query underscore areas of clinician uncertainty regarding *M. tuberculosis* infection screening and treatment in pregnant women that warrant further investigation, training, and guidelines.

Appendix 1 Additional information on network query into tuberculosis infection screening and management among pregnant migrants, Europe.

Appendix 2 Questionnaire used for network query into tuberculosis infection screening and management among pregnant migrants, Europe.
