# Supplementary material for: Query into Tuberculosis Infection Screening and Management among Pregnant Migrants, Europe
Source: Emerg Infect Dis. 2026 Mar;32(3):469–71. doi: 10.3201/eid3203.251775 (PMC13016031; doi:10.3201/eid3203.251775)
Supplement: Appendix 1 — Additional information on network query into tuberculosis infection screening and management among pregnant migrants, Europe. [file 25-1775-Techapp-s1.pdf]

Article DOI: htpps://doi.org/10.3201/eid3203.251775

EID cannot ensure accessibility for supplementary materials supplied by authors. Readers who have difficulty accessing supplementary content should contact the authors for assistance.

Query into Tuberculosis Infection Screening and Management among Pregnant Migrants, Europe

Appendix 1

Checklist for Reporting Results of Internet E-Surveys (CHERRIES) - applied to the online query

Design

Target population and sample frame

The target population consisted of clinicians involved in the care of pregnant migrant women in Europe. The query used a convenience sample, as participation was voluntary and recruitment was conducted through professional mailing lists.

Query design

This was an exploratory, web-based query.

IRB Approval and Informed Consent

IRB approval

The query was anonymous and did not collect patient-level data or personally identifiable information. According to local and institutional regulations, formal Institutional Review Board approval was not required for this type of exploratory query.

Informed consent

Information about the purpose of the query, the voluntary nature of participation, and data handling was provided on the landing page. Completion of the questionnaire was considered to imply informed consent.

Data protection

No personal identifiers were collected. Data were stored securely within the REDCap electronic data capture platform, with access restricted to the investigators.

Development and Pre-testing

Development and testing

The questionnaire was developed by members of the European Society of Clinical Microbiology and Infectious Diseases study groups involved in tuberculosis, migrant and refugee health, and maternal care. The query instrument was reviewed internally for content clarity and technical functionality before dissemination. The questionnaire included both closed-ended and open-ended questions addressing clinical practices, use of guidelines, and perceived barriers.

Recruitment Process and Description of the Sample

Open versus closed query

This was an open online query accessible through a web link and not restricted by login credentials or passwords.

Contact mode

The initial contact with potential participants was made via email through professional mailing lists. Members of the mailing lists were also encouraged to share the query link freely with colleagues who met the inclusion criteria (clinicians involved in the care of pregnant migrant women in Europe). This distribution method aimed to maximize reach but did not allow calculation of a formal denominator or response rate.

Dissemination of the query

The query was announced through mailing lists of the European Society of Clinical Microbiology and Infectious Diseases (ESCMID) Study Groups for Infections in Travelers and Migrants (ESGITM) and for Mycobacterial Infections (ESGMYC). No offline media or online banner advertising was used. The wording of the invitation described the query topic and target population.

Query Administration

Web/E-mail

The query was administered using the REDCap electronic data capture platform. Responses were captured automatically within the database.

Context

The query was distributed through professional scientific mailing lists targeting clinicians with expertise in infectious diseases, tuberculosis, migrant and refugee health, and maternal health. This context may have influenced participation and pre-selected respondents with specific professional interests.

Mandatory/voluntary

Participation was voluntary. Key items were structured to ensure completeness of essential information, while selected items were optional or conditionally displayed based on responses to preceding questions.

Time/Date

The query was open from March 4, 2025, to May 31, 2025.

Incentives

No incentives were offered.

Randomization of items or questionnaires

No randomization of items or questionnaires was implemented.

Adaptive questioning

Adaptive questioning was used, with selected items displayed conditionally based on responses to previous questions, to ensure logical consistency of the questionnaire and to avoid presenting non-applicable items to respondents.

Number of items

The questionnaire consisted of 29 items organized into 5 sequential sections addressing demographics, screening practices, screening methods, management, and training, policy, and recommendations.

Number of screens (pages)

The questionnaire was organized into 5 sequential sections within the REDCap platform.

Completeness check

Completeness checks were implemented for mandatory items before submission. Respondents could select non-response options where applicable.

Review step

Respondents were able to review and modify their answers before final submission.

Response Rates

The query was disseminated through the professional mailing lists of ESGITM and ESGMYC using an open, freely forwardable REDCap public link. At the beginning of 2025, ESGITM included 367 members and ESGMYC 219 members (potential overlap unknown). Because the link could be forwarded beyond the mailing lists and unique visits/starts were not captured, the total number of individuals reached and the denominators required for response rate calculations could not be determined. Completed questionnaires received and analyzed: n = 101

Unique site visitor

Not recorded. No IP address tracking, cookie-based identification, or other technical mechanisms were implemented to determine unique site visitors.

View rate (Ratio of unique query visitors/unique site visitors)

Not calculable. In our REDCap public-link query configuration, no visit-level analytics or unique visitor identification (eg, tokens/authentication, cookies, IP-based de-duplication) was implemented; therefore, the number of unique site visitors and the number of unique first-page query visitors were not available.

Participation rate (Ratio of unique visitors who agreed to participate/unique first query page visitors)

Not calculable. Unique first-page query visitors were not available. In addition, no discrete “agree-to-participate” step/event was implemented or logged; participation could not be counted separately from submitted questionnaires.

Completion rate (Ratio of users who finished the query/users who agreed to participate)

Not calculable. The number of users who started the query and the number who agreed to participate but did not submit (partial/non-submitted responses) were not available in the REDCap dataset (responses were recorded only upon final submission); therefore, the denominator required to calculate completion rate cannot be established. Completed questionnaires: n = 101.

Preventing Multiple Entries from the Same Individual

Cookies used

Cookies were not used to assign a unique user identifier or to prevent multiple entries.

IP check

IP address checks were not implemented to identify or restrict multiple submissions from the same user.

Log file analysis

Log file analysis was not used to detect duplicate entries.

Registration

Registration or login was not required to access the query.

Analysis

Handling of incomplete questionnaires

Only fully submitted questionnaires were included in the analysis. Because query initiations were not recorded, it was not possible to determine whether questionnaires were started but not completed.

Questionnaires submitted with an atypical timestamp

No time-based exclusion criteria were applied, and questionnaire completion time was not used as a criterion for data exclusion.

Statistical correction

No weighting, propensity scores, or other statistical adjustment methods were applied to account for the non-representative nature of the sample. Data were analyzed and reported descriptively.

Appendix Table. Characteristics of Respondents and Answers to Key Questions (N = 101).

Characteristics and questions	No. (%) respondents
Country of respondents (responses available for 98 of 101 respondents)	
Italy	29/98 (29.6)
France	13/98 (13.3)
Portugal	9/98 (9.2)
Germany	6/98 (6.1)
United Kingdom	6/98 (6.1)
Spain	4/98 (4.1)
Albania	4/98 (4.1)
Other, WHO European Region	8/98 (8.2)
Other, non–WHO European Region	19/98 (19.4)
Practices regarding TBI screening (n = 101)	
Offered to all pregnant migrants	28/101 (27.7)
Offered only to pregnant migrants with risk factors	37/101 (36.6)
Not offered to pregnant migrants	32/101 (31.7)
Other/I don't know	4/101 (4.0)
Timing of TBI screening, among respondents who reported offering TBI screening (n = 66)	
First medical visit (including antenatal visit) regardless of gestational age	44/66 (66.7)
First trimester	6/66 (9.1)
Second trimester	1/66 (1.5)
Third trimester/postpartum	0 (0.0)
Other	15/66 (22.7)
TBI diagnostic approach, among respondents who reported performing TBI screening (n = 69)†	
Sequential approach	
TST/IGRA + chest x-ray	42/69 (60.9)
TST/IGRA + Point of Care UltraSound	7/69 (10.1)
TST/IGRA + clinical assessment	26/69 (37.7)
Single test	
TST/IGRA	2/69 (2.9)
History and risk factors	2/69 (2.9)
Chest x-ray	2/69 (2.9)
No standardized approach	7/69 (10.1)
Other	2/69 (2.9)
Timing of chest x-ray when used as the primary diagnostic method (n = 18)	
First medical visit regardless of gestational age	12/18
First trimester	0 (0.0)
Second trimester	2/18
Third trimester	1/18
Postpartum	3/18
TPT practices, among respondents who reported performing TBI screening (n = 69)	
Offered during pregnancy to all cases diagnosed with TBI	21/69 (30.4)
Offered only in case of risk factors	28/69 (40.6)
Not offered during pregnancy/postponed after delivery to all	16/69 (23.2)
Other	4/69 (5.8)
Timing of TPT initiation, among respondents who reported offering TPT during pregnancy (n = 52)	
At the time of diagnosis, regardless of gestational age	28/52 (53.8)
First trimester	0 (0.0)
Second trimester	7/52 (13.5)
Depending on individual risk assessment	17/52 (32.7)
Guidelines/Reference documents followed in clinical practice, among respondents reporting use of guidelines (n = 35)†	
International guidelines	22/35 (62.9)
National guidelines	17/35 (48.6)
Local protocols	4/35 (11.4)
Major limitations of existing guidelines, among respondents reporting them (n = 70)†	
Lack of sufficient evidence for specific populations, such as pregnant women	42/70 (60.0)
Inadequate guidance on how to screen TBI during pregnancy	23/70 (32.9)
Inadequate guidance on how to treat TBI during pregnancy	25/70 (35.7)

Characteristics and questions	No. (%) respondents
Unclear recommendations on when to initiate treatment during pregnancy	26/70 (37.1)
Lack of consensus on the preferred diagnostic tests in pregnancy	11/70 (15.7)
Limited recommendations on monitoring and follow-up care during pregnancy	20/70 (28.6)
Variability in national guidelines leading to inconsistent implementation	19/70 (27.1)
Other	7/70 (10.0)
Additional resources identified to improve the management of TBI in pregnant migrant women (responses available for 99 of 101 respondents)†	
Additional training	65/99 (65.7)
Improved access to guidelines	52/99 (52.5)
Development of standardized protocols	71/99 (71.5)
Coverage of costs for TBI screening/treatment	29/99 (29.3)
Availability of cultural mediators	47/99 (47.5)
Other	1/99 (1.0)

*IGRA, Interferon Gamma Release Assay; TBI, Tuberculosis Infection; TPT, Tuberculosis Preventive Treatment; TST, Tuberculin Skin Test; WHO: World Health Organization.

†Multiple responses were allowed.
