# Supplementary material for: Query into Tuberculosis Infection Screening and Management among Pregnant Migrants, Europe
Source: Emerg Infect Dis. 2026 Mar;32(3):469–71. doi: 10.3201/eid3203.251775 (PMC13016031; doi:10.3201/eid3203.251775)
Supplement: Appendix 2 — Questionnaire used for network query into tuberculosis infection screening and management among pregnant migrants, Europe. [file 25-1775-Techapp-s2.pdf]

Article DOI: htpps://doi.org/10.3201/eid3203.251775

EID cannot ensure accessibility for supplementary materials supplied by authors. Readers who have difficulty accessing supplementary content should contact the authors for assistance.

Query into Tuberculosis Infection Screening and Management among Pregnant Migrants, Europe

Appendix 2

Questionnaire for Network Query into Tuberculosis Screening and Management among Pregnant Migrants, Europe, 2025

Network Query on Tuberculosis Infection Screening and Management among Pregnant Migrants in Europe by ESGITM and ESGMYC, 2025

Section 1: Demographics

What is your age? (Please enter your age in years)

- ◯ < 35
- ◯ 36 - 50
- ◯ 51 - 65
- ◯ >65

Which professional category best defines your role?

- ◯ Infectious Disease Specialist
- ◯ Pulmonologist
- ◯ Obstetrician/Gynecologist
- ◯ General Practitioner
- ◯ Public Health Specialist
- ◯ Midwife/Nurse
- ◯ Clinical Researcher
- ◯ Laboratory-based Researcher
- ◯ Policy Maker
- ◯ Other ______

What type of healthcare setting do you work in? Type of healthcare facility:

_____

Area of care/specific setting:

_____

Which country are you currently working in?

- ◯ Afghanistan
- ◯ Albania
- ◯ Algeria
- ◯ Andorra
- ◯ Angola
- ◯ Antigua & Barbuda
- ◯ Argentina
- ◯ Armenia
- ◯ Australia
- ◯ Austria
- ◯ Azerbaijan
- ◯ Bahamas
- ◯ Bahrain
- ◯ Bangladesh
- ◯ Barbados
- ◯ Belarus
- ◯ Belgium
- ◯ Belize
- ◯ Benin
- ◯ Bhutan
- ◯ Bolivia
- ◯ Bosnia & Herzegovina
- ◯ Botswana
- ◯ Brazil
- ◯ Brunei
- ◯ Bulgaria
- ◯ Burkina Faso
- ◯ Burundi
- ◯ Cabo Verde
- ◯ Cambodia
- ◯ Cameroon
- ◯ Canada
- ◯ Central African Republic
- ◯ Chad
- ◯ Chile
- ◯ China
- ◯ Colombia
- ◯ Comoros
- ◯ Congo
- ◯ Costa Rica
- ◯ Côte d'Ivoire
- ◯ Croatia
- ◯ Cuba
- ◯ Cyprus
- ◯ Czech Republic
- ◯ Denmark
- ◯ Djibouti
- ◯ Dominica
- ◯ Dominican Republic
- ◯ DR Congo
- ◯ Ecuador
- ◯ Egypt
- ◯ El Salvador
- ◯ Equatorial Guinea
- ◯ Eritrea
- ◯ Estonia
- ◯ Eswatini
- ◯ Ethiopia
- ◯ Fiji
- ◯ Finland
- ◯ France
- ◯ Gabon
- ◯ Gambia
- ◯ Georgia
- ◯ Germany
- ◯ Ghana
- ◯ Greece
- ◯ Grenada
- ◯ Guatemala

- ◯ Guinea
- ◯ Guinea-Bissau
- ◯ Guyana
- ◯ Haiti
- ◯ Holy See
- ◯ Honduras
- ◯ Hungary
- ◯ Iceland
- ◯ India
- ◯ Indonesia
- ◯ Iran
- ◯ Iraq
- ◯ Ireland
- ◯ Israel
- ◯ Italy
- ◯ Jamaica
- ◯ Japan
- ◯ Jordan
- ◯ Kazakhstan
- ◯ Kenya
- ◯ Kiribati
- ◯ Kuwait
- ◯ Kyrgyzstan
- ◯ Laos
- ◯ Latvia
- ◯ Lebanon
- ◯ Lesotho
- ◯ Liberia
- ◯ Libya
- ◯ Liechtenstein
- ◯ Lithuania
- ◯ Luxembourg
- ◯ Madagascar
- ◯ Malawi
- ◯ Malaysia
- ◯ Maldives
- ◯ Mali
- ◯ Malta
- ◯ Marshall Islands
- ◯ Mauritania
- ◯ Mauritius
- ◯ Mexico
- ◯ Micronesia
- ◯ Moldova
- ◯ Monaco
- ◯ Mongolia
- ◯ Montenegro
- ◯ Morocco
- ◯ Mozambique
- ◯ Myanmar
- ◯ Namibia
- ◯ Nauru
- ◯ Nepal
- ◯ Netherlands
- ◯ New Zealand
- ◯ Nicaragua
- ◯ Niger
- ◯ Nigeria
- ◯ North Korea
- ◯ North Macedonia
- ◯ Norway
- ◯ Oman
- ◯ Pakistan
- ◯ Palau
- ◯ Panama
- ◯ Papua New Guinea
- ◯ Paraguay
- ◯ Peru
- ◯ Philippines
- ◯ Poland
- ◯ Portugal

- Qatar
- Romania
- Russia
- Rwanda
- Saint Kitts & Nevis
- Saint Lucia
- Samoa
- San Marino
- Sao Tome & Principe
- Saudi Arabia
- Senegal
- Serbia
- Seychelles
- Sierra Leone
- Singapore
- Slovakia
- Slovenia
- Solomon Islands
- Somalia
- South Africa
- South Korea
- South Sudan
- Spain
- Sri Lanka
- St. Vincent & Grenadines
- State of Palestine
- Sudan
- Suriname
- Sweden
- Switzerland
- Syria
- Tajikistan
- Tanzania
- Thailand
- Timor-Leste
- Togo
- Tonga
- Trinidad & Tobago
- Tunisia
- Turkey
- Turkmenistan
- Tuvalu
- Uganda
- Ukraine
- United Arab Emirates
- United Kingdom
- United States
- Uruguay
- Uzbekistan
- Vanuatu
- Venezuela
- Vietnam
- Yemen
- Zambia
- Zimbabwe

How long have you been involved in the management of TBI in pregnant migrants?

- Less than 1 year
- 1-3 years
- 3-5 years
- More than 5 years

Section 2: TBI Screening Practices

Is TBI screening performed for pregnant migrants in your healthcare centre?

- ◯ Yes, screening is routinely offered to all pregnant migrant women
- ◯ Yes, screening is offered to pregnant migrants under specific conditions (e.g. recent exposure, risk factors)
- ◯ No, TBI screening is not offered to pregnant migrant women
- ◯ Other ______
- ◯ I don't know

If TBI screening during pregnancy is offered to women with specific risk factors, which of the following risk factors are considered?

- ☐ Recent contact with an active TB case
- ☐ Recent migration from a high TB-burden country (≤ 5 years)
- ☐ HIV infection
- ☐ Immunosuppression (e.g. due to medications or medical conditions)
- ☐ Diabetes mellitus
- ☐ Malnutrition
- ☐ Smoking history
- ☐ Homelessness
- ☐ Other ______

Is TBI screening performed more than once during the same pregnancy?

- ◯ Yes, TBI screening is repeated if the first test was negative
- ◯ Yes, TBI screening is repeated under specific conditions (e.g., high-risk exposure)
- ◯ No, TBI screening is performed only once per pregnancy
- ◯ Other ______
- ◯ I don't know

At what stage of pregnancy or perinatal care is TBI screening typically performed?

- ◯ During the first antenatal care visit, regardless of the gestational age
- ◯ First trimester
- ◯ Second trimester
- ◯ Third trimester
- ◯ Postpartum
- ◯ Other ______

Section 3: TBI Screening Methods

What screening methods are primarily used to screen TBI in pregnant migrants?

- ☐ Tuberculin skin test (TST)
- ☐ Interferon-gamma release assays (IGRAs)
- ☐ Chest X-ray
- ☐ Point-of-care ultrasound
- ☐ Symptom-based screening only
- ☐ Other ______

How is TBI typically diagnosed in pregnant migrants who underwent screening in your centre?	☐ Based on positive TST or IGRA result, followed by Chest X-ray to rule out active TB<br>☐ Based on positive TST or IGRA result, followed by Point-of-care ultrasound to rule out active TB<br>☐ Based on positive TST or IGRA result, followed by clinical assessment to rule out active TB<br>☐ Based on positive TST or IGRA result only<br>☐ Based on clinical risk factors and history only<br>☐ Based on chest X-ray findings only (if performed due to TB suspicion)<br>☐ No standardized diagnostic protocol is followed<br>☐ Other ______
At what stage of pregnancy or perinatal care do you typically perform a Chest X-ray?	○ At the time of initial assessment, regardless of gestational age<br>○ First trimester<br>○ Second trimester<br>○ Third trimester<br>○ Postpartum
Do you encounter any barriers when performing TBI screening on pregnant migrants?	○ Yes<br>○ No
Please select all that apply:	☐ Patient compliance ______<br>☐ Financial or healthcare system barriers ______<br>☐ Issues concerning Chest-X ray _______<br>☐ National guidelines and protocols ______<br>☐ Other ______

Section 4: TBI Management

How is TBI managed in pregnant migrant women in your healthcare centre?	○ Preventive treatment is offered during pregnancy to all migrants diagnosed with TBI<br>○ Preventive treatment is not offered during pregnancy or is delayed until after delivery<br>○ Preventive treatment is offered only under specific conditions (e.g., recent exposure, risk factors)<br>○ Other ______
If TBI preventive treatment during pregnancy is restricted to women with specific risk factors, which of the following risk factors are considered?	☐ Recent contact with an active TB case<br>☐ Recent migration from a high TB-burden country (≤ 5 years)<br>☐ HIV infection<br>☐ Immunosuppression (e.g., due to medications or medical conditions)<br>☐ Diabetes mellitus<br>☐ Malnutrition<br>☐ Smoking history<br>☐ Homelessness<br>☐ Other ______
If TBI preventive treatment is initiated during pregnancy, at what trimester is it usually started?	○ Once the diagnosis of TBI is made, regardless of the gestational age<br>○ First trimester<br>○ Second trimester<br>○ Third trimester<br>○ It depends on individual risk assessment<br>○ Other ______

If TBI preventive treatment is offered during pregnancy, which regimen is most commonly used?	◯ Isoniazid (INH) monotherapy<br>◯ Rifampicin (RIF) monotherapy<br>◯ Combination therapy INH + RIF<br>◯ Combination therapy INH + Rifapentine<br>◯ Other ______
Do you encounter any challenges in the decision-making process or in prescribing TBI treatment for pregnant migrants, including ensuring patient adherence?	◯ Yes<br>◯ No
Please select all that apply	☐ Healthcare Provider-Related Factors ______<br>☐ Patient-Related Factors ______<br>☐ Healthcare System Barriers ______<br>☐ Guideline and Policy Barriers ______<br>☐ Other ______
What is the typical adherence rate to TBI preventive treatment among pregnant migrant women in your centre?	◯ High adherence (above 80%)<br>◯ Moderate adherence (50-80%)<br>◯ Low adherence (below 50%)<br>◯ I don't know

Section 5: Training, Policy, and Recommendations

Do you follow or are you aware of any specific guidelines or reference documents regarding TBI screening in pregnant women?	◯ Yes<br>◯ No<br>◯ Not sure
Which guidelines or reference documents do you follow for TBI screening in pregnant women?	☐ International guidelines<br>☐ National guidelines<br>☐ Local protocols<br>☐ Other ______

Please provide the following information for each: Title, DOI (if available), URL or link

Do you believe that existing guidelines for TBI screening in pregnant women are adequate?	◯ Yes<br>◯ No<br>◯ Not sure
What do you perceive as the major limitations of the existing guidelines?	☐ Lack of sufficient evidence for specific populations, such as pregnant women<br>☐ Inadequate guidance on how to screen TBI during pregnancy<br>☐ Inadequate guidance on how to treat TBI during pregnancy<br>☐ Unclear recommendations on when to initiate treatment during pregnancy<br>☐ Lack of consensus on the preferred diagnostic tests in pregnancy<br>☐ Limited recommendations on monitoring and follow-up care during pregnancy<br>☐ Variability in national guidelines leading to inconsistent implementation<br>☐ Other (please specify)

Which specific training have you received on TBI screening and management in pregnant women?	☐ Extensive specialized training (e.g., advanced courses, certified programs, hands-on workshops)<br>☐ Basic introductory training (e.g., brief workshops, online modules, lectures)<br>☐ Training on national or international guidelines<br>☐ Training on institutional or local protocols<br>☐ On-the-job training or informal learning<br>☐ No specific training
What additional resources would improve the management of TBI in pregnant migrant women in your setting?	☐ Additional training on TBI screening and management for vulnerable populations such as pregnant migrants<br>☐ Improved access to relevant guidelines or protocols<br>☐ Development of standardized protocols for TBI in pregnancy<br>☐ Coverage of costs for screening and treatment<br>☐ Availability of cultural mediators<br>☐ Other ______
